# *fac*-(2-Amido­ethyl-κ^2^ *C* ^1^,*O*)aqua­tri­chlorido­tin(IV) 1,4,7,10,13,16-hexa­oxacyclo­octa­decane (2/1)

**DOI:** 10.1107/S1600536810005908

**Published:** 2010-02-20

**Authors:** Solange M. S. V. Wardell, William T. A. Harrison, Edward R. T. Tiekink, Geraldo M. de Lima, James L. Wardell

**Affiliations:** a CHEMSOL, 1 Harcourt Road, Aberdeen AB15 5NY, Scotland; b Department of Chemistry, University of Aberdeen, Meston Walk, Old Aberdeen, AB24 3NY, Scotland; c Department of Chemistry, University of Malaya, 50603 Kuala Lumpur, Malaysia; d Departamento de Quimica, ICEx, Universidade Federal de Minas Gerais, 31270-901 Belo Horizonte, MG, Brazil; e Centro de Desenvolvimento Tecnológico em Saúde (CDTS), Fundação Oswaldo Cruz (FIOCRUZ), Casa Amarela, Campus de Manguinhos, Av. Brasil 4365, 21040-900, Rio de Janeiro, RJ, Brazil

## Abstract

The asymmetric unit of the title compound, [Sn(C_3_H_6_NO)Cl_3_(H_2_O)]_2_·C_12_H_24_O_6_, comprises a six-coordinate tin complex and a 18-crown-6 mol­ecule, the latter disposed about a centre of inversion. The tin atom is coordinated by three Cl atoms, that define a facial arrangement, a chelating *C*-,*O*- ligand, and a water mol­ecule. The resulting CCl_3_O_2_ donor set defines a distorted octa­hedral geometry. The tin-bound aqua ligand forms O—H⋯O hydrogen bonds to the centrosymmetric 18-crown-6 mol­ecule, resulting in a tri-mol­ecular aggregate. These assemble into a supra­molecular chain along the *a* axis being connected by N—H⋯O hydrogen bonds.

## Related literature

For background to amido­ethyl tin compounds, see: Hutton & Oakes (1976 ▶). For the use of organotin compounds as PVC stabilisers, see: Lanigen & Weinberg (1976 ▶). For the crystal structures of amido­ethyl­tin compounds, see: Harrison *et al.* (1979 ▶); Tiekink *et al.* (2006 ▶). For the crystal structures of alkyl­oxycarbonyl­ethyl­tin compounds, see: de Lima *et al.* (2009 ▶); Milne *et al.* (2005 ▶). For a review on tin–crown ether compounds, see: Cusack & Smith (1990 ▶). For related structures of organotin(IV) and tin(IV) halide complexes with crown ethers, see: Cusack *et al.* (1983 ▶); Amini *et al.* (1984 ▶, 2002 ▶); Russo *et al.* (1984 ▶); Valle *et al.* (1984 ▶, 1985 ▶); Rivarola *et al.* (1986 ▶); Bott *et al.* (1987 ▶); Mitra *et al.* (1993 ▶); Yap *et al.* (1996 ▶); Wolff *et al.* (2009 ▶).
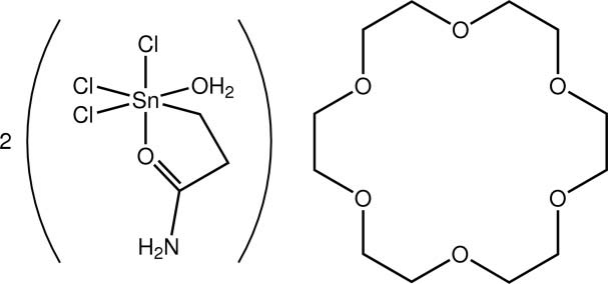


## Experimental

### 

#### Crystal data

[Sn(C_3_H_6_NO)Cl_3_(H_2_O)]_2_·C_12_H_24_O_6_
*M* *_r_* = 894.64
Monoclinic, 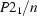

*a* = 10.1260 (2) Å
*b* = 10.0893 (3) Å
*c* = 15.8229 (4) Å
β = 105.814 (2)°
*V* = 1555.35 (7) Å^3^
*Z* = 2
Mo *K*α radiation
μ = 2.17 mm^−1^
*T* = 120 K
0.20 × 0.18 × 0.02 mm

#### Data collection

Nonius KappaCCD area-detector diffractometer
Absorption correction: multi-scan (*SADABS*; Sheldrick, 2007 ▶) *T*_min_ = 0.638, *T*_max_ = 0.746
19526 measured reflections
3555 independent reflections
2981 reflections with *I* > 2σ(*I*)
*R*_int_ = 0.062

#### Refinement

*R*[*F* ^2^ > 2σ(*F* ^2^)] = 0.030
*wR*(*F* ^2^) = 0.090
*S* = 1.12
3555 reflections
184 parameters
6 restraints
H atoms treated by a mixture of independent and constrained refinement
Δρ_max_ = 0.81 e Å^−3^
Δρ_min_ = −1.31 e Å^−3^

### 

Data collection: *COLLECT* (Hooft, 1998 ▶); cell refinement: *DENZO* (Otwinowski & Minor, 1997 ▶) and *COLLECT*; data reduction: *DENZO* and *COLLECT*; program(s) used to solve structure: *SHELXS97* (Sheldrick, 2008 ▶); program(s) used to refine structure: *SHELXL97* (Sheldrick, 2008 ▶); molecular graphics: *DIAMOND* (Brandenburg, 2006 ▶); software used to prepare material for publication: *publCIF* (Westrip, 2010 ▶).

## Supplementary Material

Crystal structure: contains datablocks general, I. DOI: 10.1107/S1600536810005908/lh2997sup1.cif

Structure factors: contains datablocks I. DOI: 10.1107/S1600536810005908/lh2997Isup2.hkl

Additional supplementary materials:  crystallographic information; 3D view; checkCIF report

## Figures and Tables

**Table 1:** Hydrogen-bond geometry (Å, °)

*D*—H⋯*A*	*D*—H	H⋯*A*	*D*⋯*A*	*D*—H⋯*A*
O1w—H1w⋯O4	0.84 (2)	1.96 (2)	2.784 (3)	166 (3)
O1w—H2w⋯O2	0.84 (3)	2.01 (3)	2.839 (3)	169 (4)
N1—H1n⋯O3^i^	0.88 (3)	2.51 (3)	3.061 (4)	121 (2)
N1—H2n⋯Cl1^ii^	0.88 (3)	2.68 (3)	3.516 (3)	161 (3)

Symmetry codes: (i) 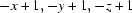; (ii) 
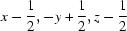
.

**Fig. 1.**
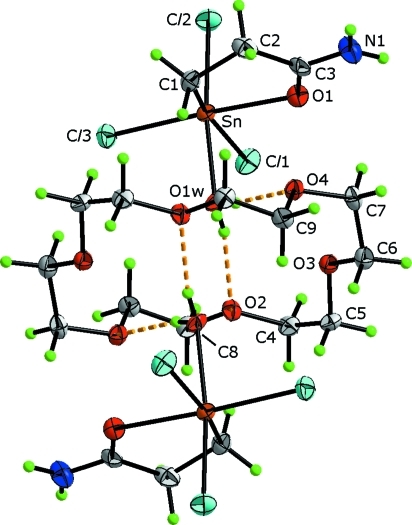
The molecular structure of a tri-molecular aggregate in (I) showing the atom-labelling scheme and displacement ellipsoids at the 50% probability level. Dashed lines indicate hydrogen bonds.

**Fig. 2.**
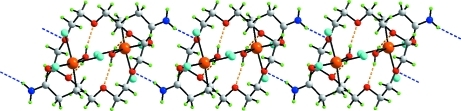
A view of a supramolecular chain in (I), aligned along the a axis, whereby the tri-molecular aggregates sustained by O–H···O hydrogen bonds (orange dashed lines) illustrated in Fig. 1, are connected via N–H···O hydrogen bonds (blue dashed lines). Colour code: Sn, orange; Cl, cyan; O, red; N, blue; C, grey; and H, green.

**Fig. 3.**
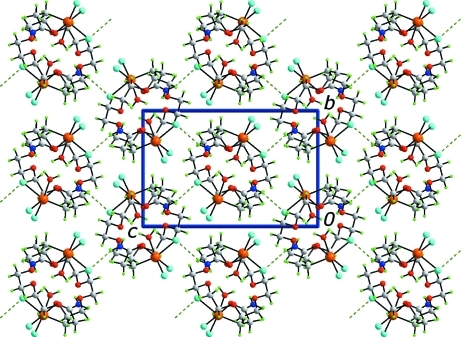
View in projection down the a axis of the unit cell contents in (I). The N–H···Cl interactions connecting the supramolecular chains illustrated in Fig. 2 are shown as green dashed lines. Colour code: Sn, orange; Cl, cyan; O, red; N, blue; C, grey; and H, green.

## supplementary crystallographic information

### Comment

Functionally substituted-alkyl-tin compounds, X_3_SnCR_2_CH_2_COY and X_2_Sn(CR_2_CH_2_COY)_2_ (X = halide, R = H or alkyl; Y = OR', R' or NH_2_, R' = alkyl or aryl), are available from reactions, first reported in the 1970's (Hutton & Oakes, 1976), of R_2_C=CHCOY, HX and SnX_2_ (for X_3_SnCR_2_CH_2_COY compounds) or HX and tin (for X_2_Sn(CR_2_CH_2_COY)_2_ compounds). Original interest with these compounds was primarily concerned with their industrial potential as precursors of PVC stabilizers (Lanigen & Weinberg, 1976) but also with regard to their coordination chemistry. Although the potential for use in PVC stabilization has not been realized commercially, the interest in the coordination chemistry, especially of compounds containing SnCR_2_CH_2_CO_2_R moieties, has been maintained over the succeeding decades: of particular interest has been the coordination modes of the CR_2_CH_2_COY ligands (de Lima *et al.*, 2009; Milne *et al.*, 2005; Harrison *et al.*, 1979). Much less study has been made of amidoethyl-tin species. *i*.*e*., tin compounds containing the CH_2_CH_2_CONH_2_ group. Only two structures of amidoethyltin derivatives have been previously reported, namely of (H_2_NHCOCH_2_CH_2_-*C*,*O*)_2_SnCl_2_ (Harrison *et al.*, 1979) and (H_2_NCOCH_2_CH_2_-*C*,*O*)(ClCH_2_CH_2_CONH_2_-*O*)SnCl_3_ (Tiekink *et al.*, 2006). We now wish to report the crystal structure of *fac*-aqua-trichloro(2-amidoethyl-*C*,*O*)tin 1,4,7,10,13,16-hexaoxacyclooctadecane (2/1), (I). Crown ether complexes of tin and organotin halides have been variously reported (Cusack *et al.* 1983; Amini *et al.*, 1984; Valle *et al.*, 1984; Russo *et al.*, 1984; Valle *et al.*, 1985; Rivarola *et al.*, 1986; Bott *et al.*, 1987; Cusack & Smith, 1990; Mitra *et al.*, 1993; Yap *et al.*, 1996; Amini *et al.*, 2002; Wolff *et al.*, 2009).

The asymmetric unit of (I) comprises a tin complex and half a 18-crown-6 molecule as the latter is situated about a centre of inversion, Fig. 1. The tin atom is coordinated by three Cl atoms, that define a facial arrangement, a *C*-,*O*-chelating ligand, and an aqua ligand. The resulting CCl_3_O_2_ donor set defines a distorted octahedral geometry. The Cl atoms *trans* to O-donors form longer Sn–Cl bond distances [Sn–Cl2 = 2.4208 (9) and Sn–Cl3 = 2.4329 (9) Å] than the Cl atom *trans* to the C1 atom [Sn–Cl1 = 2.3800 (9) Å]. The five-membered SnC_3_O chelate ring is not planar as seen in the values of the Sn–C1–C2–C3 and C1–C2–C3–O1 torsion angles of 24.0 (4) and -15.9 (5) °, respectively.

The components of the structure are connected via O_aqua_–H···O_ether_ hydrogen bonds to form a tri-molecular aggregate, Table 1 and Fig. 1. These in turn are connected via N–H···O_ether_ hydrogen bonds so that all ether-O atoms participate in hydrogen bonding interactions, Table 1. The resulting supramolecular aggregate is a linear chain formed along the *a* axis, Fig. 2. These are connected into the 3-D crystal structure via N–H···Cl interactions, Fig. 3.

### Experimental

The reaction between SnCl_2_, H_2_C═CHCONH_2_ and gaseous HCl in diethyl ether, as previously reported (Tiekink *et al.*, 2006), produced (H_2_NCOCH_2_CH_2_-*C*,*O*)(ClCH_2_CH_2_CONH_2_-*O*)SnCl_3_. Solutions of 1,4,7,10,13,16-hexaoxacyclooctadecane (18-crown-6) (0.26 g, 1 mmol) in EtOH (15 ml) and (H_2_NCOCH_2_CH_2_-*C*,*O*)(ClCH_2_CH_2_CONH_2_-*O*)SnCl_3_ (0.40 g, 1 mmol) in EtOH (20 ml) were mixed and gently heated for 15 min. The reaction mixture was cooled and maintained at room temperature. The crystals which slowly appeared on evaporation of the solvent were harvested after 1 week. M.pt.: partial sublimation at 463 K with complete melting at 469-471 K. IR (KBr, cm^-1^): 3441, 3349, 3273, 2919(br), 1650, 1573, 1456, 1352, 1297, 1254, 1094, 1037, 958, 915, 844, 702, 564.

### Refinement

The C-bound H atoms were geometrically placed (C–H = 0.99 Å) and refined as riding with *U_iso_*(H) = 1.2*U_eq_*(C). The O- and N- bound and H atoms were located from difference maps and refined with O–H = 0.84±0.01 Å and N–H = 0.88±0.01 Å, and with *U*_iso_(H) = 1.5*U_eq_*(O, N). The maximum and minimum residual electron density peaks of 0.81 and 1.31 e Å^-3^, respectively, were located 1.29 Å and 0.79 Å from the H4a and Sn atoms, respectively.

### Crystal data

**Table:**

[Sn(C_3_H_6_NO)Cl_3_(H_2_O)]_2_·C_12_H_24_O_6_	*F*(000) = 888
*M**_r_* = 894.64	*D*_x_ = 1.910 Mg m^−^^3^
Monoclinic, *P*2_1_/*n*	Mo *K*α radiation, λ = 0.71073 Å
Hall symbol: -P 2yn	Cell parameters from 9442 reflections
*a* = 10.1260 (2) Å	θ = 2.9–27.5°
*b* = 10.0893 (3) Å	µ = 2.17 mm^−^^1^
*c* = 15.8229 (4) Å	*T* = 120 K
β = 105.814 (2)°	Prism, colourless
*V* = 1555.35 (7) Å^3^	0.20 × 0.18 × 0.02 mm
*Z* = 2	

### Data collection

**Table:**

Nonius KappaCCD area-detector diffractometer	3555 independent reflections
Radiation source: Enraf Nonius FR591 rotating anode	2981 reflections with *I* > 2σ(*I*)
10 cm confocal mirrors	*R*_int_ = 0.062
Detector resolution: 9.091 pixels mm^-1^	θ_max_ = 27.5°, θ_min_ = 3.0°
φ and ω scans	*h* = −13→12
Absorption correction: multi-scan (*SADABS*; Sheldrick, 2007)	*k* = −13→13
*T*_min_ = 0.638, *T*_max_ = 0.746	*l* = −20→20
19526 measured reflections	

### Refinement

**Table:**

Refinement on *F*^2^	Primary atom site location: structure-invariant direct methods
Least-squares matrix: full	Secondary atom site location: difference Fourier map
*R*[*F*^2^ > 2σ(*F*^2^)] = 0.030	Hydrogen site location: inferred from neighbouring sites
*wR*(*F*^2^) = 0.090	H atoms treated by a mixture of independent and constrained refinement
*S* = 1.12	*w* = 1/[σ^2^(*F*_o_^2^) + (0.0486*P*)^2^] where *P* = (*F*_o_^2^ + 2*F*_c_^2^)/3
3555 reflections	(Δ/σ)_max_ = 0.002
184 parameters	Δρ_max_ = 0.81 e Å^−^^3^
6 restraints	Δρ_min_ = −1.31 e Å^−^^3^

### Special details

**Table:**

Geometry. All s.u.'s (except the s.u. in the dihedral angle between two l.s. planes) are estimated using the full covariance matrix. The cell s.u.'s are taken into account individually in the estimation of s.u.'s in distances, angles and torsion angles; correlations between s.u.'s in cell parameters are only used when they are defined by crystal symmetry. An approximate (isotropic) treatment of cell s.u.'s is used for estimating s.u.'s involving l.s. planes.
Refinement. Refinement of *F*^2^ against ALL reflections. The weighted *R*-factor wR and goodness of fit *S* are based on *F*^2^, conventional *R*-factors *R* are based on *F*, with *F* set to zero for negative *F*^2^. The threshold expression of *F*^2^ > 2σ(*F*^2^) is used only for calculating *R*-factors(gt) etc. and is not relevant to the choice of reflections for refinement. *R*-factors based on *F*^2^ are statistically about twice as large as those based on *F*, and *R*- factors based on ALL data will be even larger.

### Fractional atomic coordinates and isotropic or equivalent isotropic displacement parameters (Å^2^)

**Table:**

	*x*	*y*	*z*	*U*_iso_*/*U*_eq_	
Sn	0.78251 (2)	0.23444 (2)	0.571426 (15)	0.01204 (10)	
Cl1	0.77631 (9)	0.37992 (9)	0.68869 (6)	0.0199 (2)	
Cl2	0.64299 (9)	0.07335 (9)	0.62109 (6)	0.0231 (2)	
Cl3	0.99961 (8)	0.13617 (9)	0.65225 (6)	0.0200 (2)	
O1	0.5890 (2)	0.3331 (2)	0.49675 (15)	0.0146 (5)	
O1W	0.8950 (2)	0.4054 (2)	0.53264 (16)	0.0139 (5)	
H1W	0.859 (3)	0.436 (4)	0.4824 (12)	0.021*	
H2W	0.922 (4)	0.471 (2)	0.565 (2)	0.021*	
N1	0.4259 (3)	0.3257 (3)	0.3687 (2)	0.0217 (7)	
H1N	0.377 (3)	0.386 (3)	0.387 (2)	0.033*	
H2N	0.396 (4)	0.290 (4)	0.3164 (14)	0.033*	
C1	0.7646 (3)	0.1561 (3)	0.4426 (2)	0.0155 (7)	
H1A	0.8272	0.2038	0.4148	0.019*	
H1B	0.7894	0.0610	0.4465	0.019*	
C2	0.6165 (3)	0.1739 (4)	0.3884 (2)	0.0191 (8)	
H2A	0.6154	0.1925	0.3267	0.023*	
H2B	0.5663	0.0899	0.3890	0.023*	
C3	0.5422 (3)	0.2844 (3)	0.4209 (2)	0.0157 (7)	
O2	1.0228 (2)	0.6214 (2)	0.63876 (15)	0.0146 (5)	
O3	0.7765 (2)	0.6756 (2)	0.51861 (15)	0.0146 (5)	
O4	0.7319 (2)	0.4951 (2)	0.37156 (15)	0.0140 (5)	
C4	0.9395 (4)	0.7297 (3)	0.6529 (3)	0.0172 (8)	
H4A	0.9985	0.8011	0.6861	0.021*	
H4B	0.8774	0.6993	0.6877	0.021*	
C5	0.8571 (4)	0.7812 (3)	0.5659 (3)	0.0175 (8)	
H5A	0.7967	0.8541	0.5745	0.021*	
H5B	0.9190	0.8161	0.5323	0.021*	
C6	0.7026 (3)	0.7148 (4)	0.4323 (2)	0.0165 (7)	
H6A	0.7659	0.7537	0.4012	0.020*	
H6B	0.6326	0.7820	0.4349	0.020*	
C7	0.6351 (3)	0.5932 (3)	0.3852 (2)	0.0156 (7)	
H7A	0.5772	0.5525	0.4195	0.019*	
H7B	0.5741	0.6196	0.3274	0.019*	
C8	1.1285 (3)	0.5885 (3)	0.7162 (2)	0.0148 (7)	
H8A	1.0876	0.5676	0.7648	0.018*	
H8B	1.1917	0.6646	0.7342	0.018*	
C9	0.7943 (3)	0.5295 (3)	0.3033 (2)	0.0149 (7)	
H9A	0.8578	0.6050	0.3223	0.018*	
H9B	0.7227	0.5562	0.2498	0.018*	

### Atomic displacement parameters (Å^2^)

**Table:**

	*U*^11^	*U*^22^	*U*^33^	*U*^12^	*U*^13^	*U*^23^
Sn	0.01329 (15)	0.01173 (14)	0.01132 (15)	−0.00019 (8)	0.00373 (10)	0.00053 (9)
Cl1	0.0273 (5)	0.0190 (5)	0.0138 (5)	0.0020 (4)	0.0062 (4)	−0.0027 (3)
Cl2	0.0223 (4)	0.0194 (5)	0.0303 (5)	−0.0025 (4)	0.0118 (4)	0.0071 (4)
Cl3	0.0157 (4)	0.0195 (5)	0.0227 (5)	0.0027 (3)	0.0018 (4)	0.0049 (4)
O1	0.0164 (12)	0.0174 (13)	0.0101 (12)	0.0007 (10)	0.0036 (10)	−0.0028 (10)
O1W	0.0189 (12)	0.0094 (12)	0.0121 (13)	0.0002 (10)	0.0023 (10)	0.0026 (10)
N1	0.0165 (15)	0.0297 (19)	0.0176 (17)	0.0027 (14)	0.0023 (13)	−0.0074 (14)
C1	0.0176 (17)	0.0149 (18)	0.0152 (18)	0.0015 (14)	0.0062 (14)	−0.0013 (14)
C2	0.0167 (18)	0.024 (2)	0.017 (2)	−0.0039 (15)	0.0054 (15)	−0.0063 (16)
C3	0.0115 (16)	0.0186 (18)	0.0194 (19)	−0.0020 (14)	0.0084 (14)	0.0025 (15)
O2	0.0135 (11)	0.0160 (12)	0.0129 (13)	0.0015 (9)	0.0011 (9)	−0.0024 (9)
O3	0.0142 (12)	0.0128 (12)	0.0154 (13)	0.0005 (10)	0.0017 (10)	−0.0002 (10)
O4	0.0149 (11)	0.0138 (12)	0.0135 (12)	0.0011 (9)	0.0044 (9)	0.0023 (9)
C4	0.0170 (18)	0.0160 (18)	0.0194 (19)	−0.0013 (14)	0.0060 (15)	−0.0088 (14)
C5	0.0166 (18)	0.0110 (17)	0.025 (2)	−0.0019 (14)	0.0050 (15)	−0.0037 (15)
C6	0.0166 (18)	0.0162 (18)	0.017 (2)	0.0029 (14)	0.0051 (15)	0.0022 (14)
C7	0.0129 (16)	0.0199 (18)	0.0147 (18)	0.0032 (14)	0.0052 (14)	0.0029 (14)
C8	0.0176 (17)	0.0154 (17)	0.0110 (17)	0.0003 (14)	0.0029 (14)	−0.0008 (14)
C9	0.0155 (17)	0.0174 (18)	0.0128 (18)	−0.0002 (14)	0.0058 (14)	0.0035 (14)

### Geometric parameters (Å, °)

**Table:**

Sn—C1	2.147 (3)	O3—C6	1.423 (4)
Sn—O1	2.228 (2)	O3—C5	1.424 (4)
Sn—O1W	2.243 (2)	O4—C9	1.434 (4)
Sn—Cl1	2.3800 (9)	O4—C7	1.450 (4)
Sn—Cl2	2.4208 (9)	C4—C5	1.496 (5)
Sn—Cl3	2.4329 (9)	C4—H4A	0.9900
O1—C3	1.263 (4)	C4—H4B	0.9900
O1W—H1W	0.84 (2)	C5—H5A	0.9900
O1W—H2W	0.84 (3)	C5—H5B	0.9900
N1—C3	1.309 (5)	C6—C7	1.500 (5)
N1—H1N	0.88 (3)	C6—H6A	0.9900
N1—H2N	0.88 (3)	C6—H6B	0.9900
C1—C2	1.522 (5)	C7—H7A	0.9900
C1—H1A	0.9900	C7—H7B	0.9900
C1—H1B	0.9900	C8—C9^i^	1.502 (5)
C2—C3	1.512 (5)	C8—H8A	0.9900
C2—H2A	0.9900	C8—H8B	0.9900
C2—H2B	0.9900	C9—C8^i^	1.502 (5)
O2—C8	1.429 (4)	C9—H9A	0.9900
O2—C4	1.435 (4)	C9—H9B	0.9900
			
C1—Sn—O1	80.00 (11)	C6—O3—C5	111.8 (3)
C1—Sn—O1W	86.63 (11)	C9—O4—C7	113.6 (2)
O1—Sn—O1W	87.14 (8)	O2—C4—C5	108.9 (3)
C1—Sn—Cl1	162.49 (10)	O2—C4—H4A	109.9
O1—Sn—Cl1	86.08 (6)	C5—C4—H4A	109.9
O1W—Sn—Cl1	82.09 (6)	O2—C4—H4B	109.9
C1—Sn—Cl2	98.92 (10)	C5—C4—H4B	109.9
O1—Sn—Cl2	88.03 (6)	H4A—C4—H4B	108.3
O1W—Sn—Cl2	171.91 (6)	O3—C5—C4	108.7 (3)
Cl1—Sn—Cl2	91.10 (3)	O3—C5—H5A	110.0
C1—Sn—Cl3	100.34 (10)	C4—C5—H5A	110.0
O1—Sn—Cl3	177.30 (6)	O3—C5—H5B	110.0
O1W—Sn—Cl3	90.20 (6)	C4—C5—H5B	110.0
Cl1—Sn—Cl3	93.07 (3)	H5A—C5—H5B	108.3
Cl2—Sn—Cl3	94.55 (3)	O3—C6—C7	107.4 (3)
C3—O1—Sn	112.2 (2)	O3—C6—H6A	110.2
Sn—O1W—H1W	115 (3)	C7—C6—H6A	110.2
Sn—O1W—H2W	123 (3)	O3—C6—H6B	110.2
H1W—O1W—H2W	106 (4)	C7—C6—H6B	110.2
C3—N1—H1N	120 (2)	H6A—C6—H6B	108.5
C3—N1—H2N	119 (2)	O4—C7—C6	113.4 (3)
H1N—N1—H2N	120.6 (19)	O4—C7—H7A	108.9
C2—C1—Sn	107.9 (2)	C6—C7—H7A	108.9
C2—C1—H1A	110.1	O4—C7—H7B	108.9
Sn—C1—H1A	110.1	C6—C7—H7B	108.9
C2—C1—H1B	110.1	H7A—C7—H7B	107.7
Sn—C1—H1B	110.1	O2—C8—C9^i^	108.6 (3)
H1A—C1—H1B	108.4	O2—C8—H8A	110.0
C3—C2—C1	113.6 (3)	C9^i^—C8—H8A	110.0
C3—C2—H2A	108.9	O2—C8—H8B	110.0
C1—C2—H2A	108.9	C9^i^—C8—H8B	110.0
C3—C2—H2B	108.9	H8A—C8—H8B	108.4
C1—C2—H2B	108.9	O4—C9—C8^i^	108.9 (3)
H2A—C2—H2B	107.7	O4—C9—H9A	109.9
O1—C3—N1	121.1 (3)	C8^i^—C9—H9A	109.9
O1—C3—C2	121.2 (3)	O4—C9—H9B	109.9
N1—C3—C2	117.7 (3)	C8^i^—C9—H9B	109.9
C8—O2—C4	112.2 (3)	H9A—C9—H9B	108.3
			
C1—Sn—O1—C3	12.0 (2)	Sn—O1—C3—C2	−1.3 (4)
O1W—Sn—O1—C3	99.0 (2)	C1—C2—C3—O1	−15.9 (5)
Cl1—Sn—O1—C3	−178.7 (2)	C1—C2—C3—N1	165.8 (3)
Cl2—Sn—O1—C3	−87.5 (2)	C8—O2—C4—C5	165.3 (3)
O1—Sn—C1—C2	−18.7 (2)	C6—O3—C5—C4	−175.7 (3)
O1W—Sn—C1—C2	−106.4 (2)	O2—C4—C5—O3	57.7 (4)
Cl1—Sn—C1—C2	−56.6 (4)	C5—O3—C6—C7	173.4 (3)
Cl2—Sn—C1—C2	67.6 (2)	C9—O4—C7—C6	−75.0 (4)
Cl3—Sn—C1—C2	164.0 (2)	O3—C6—C7—O4	−65.3 (3)
Sn—C1—C2—C3	24.0 (4)	C4—O2—C8—C9^i^	177.2 (3)
Sn—O1—C3—N1	177.0 (3)	C7—O4—C9—C8^i^	−169.7 (3)

Symmetry codes: (i) −*x*+2, −*y*+1, −*z*+1.

### Hydrogen-bond geometry (Å, °)

**Table:**

*D*—H···*A*	*D*—H	H···*A*	*D*···*A*	*D*—H···*A*
O1w—H1w···O4	0.84 (2)	1.96 (2)	2.784 (3)	166 (3)
O1w—H2w···O2	0.84 (3)	2.01 (3)	2.839 (3)	169 (4)
N1—H1n···O3^ii^	0.88 (3)	2.51 (3)	3.061 (4)	121 (2)
N1—H2n···Cl1^iii^	0.88 (3)	2.68 (3)	3.516 (3)	161 (3)

Symmetry codes:  (ii) −*x*+1, −*y*+1, −*z*+1; (iii) *x*−1/2, −*y*+1/2, *z*−1/2.
